# Glutaraldehyde-Polymerized Hemerythrin: Evaluation of Performance as an Oxygen Carrier in Hemorrhage Models

**DOI:** 10.1155/2022/2209101

**Published:** 2022-12-30

**Authors:** Anca D. Stoica, Vlad-Al. Toma, Ioana Roman, Bogdan Sevastre, Florina Scurtu, Radu Silaghi-Dumitrescu

**Affiliations:** ^1^Department of Molecular Biology and Biotechnology, Babeș-Bolyai University, Cluj-Napoca, Romania; ^2^National Institute for Research and Development of Isotopic and Molecular Technologies, Cluj-Napoca 400293, Romania; ^3^Institute of Biological Research, Cluj-Napoca 400113, Branch of NIRDBS Bucharest, Romania; ^4^Department of Pathophysiology, University of Agricultural Sciences and Veterinary Medicine, Cluj-Napoca 400372, Romania; ^5^Faculty of Chemistry and Chemical Engineering, Babeș-Bolyai University, Cluj-Napoca 400028, Romania

## Abstract

Hemoglobin-based oxygen carriers (HBOCs) have been proposed and tested for several decades for the treatment of hemorrhage. We have previously proposed replacing hemoglobin (Hb) in HBOC with the oxygen-carrying protein hemerythrin (Hr), from marine worms, showing that Hr-based derivatives can perform at least as well or even better than Hb-based HBOC in a range of in vitro assays involving oxidative and nitrosative stress as well as in top-up animal models, where small amounts of Hr- or Hb-HBOC were injected into rats. Here, these experiments are extended to a hemorrhage experiment, in which Hr polymerized with glutaraldehyde, alone or conjugated with human serum albumin, is administered after a loss of 20–30% blood volume. The performance of these preparations is compared with that of Hb-based HBOC measured under the same conditions. Polymerized Hr is found to decrease the survival rate and can hence cannot be used as an oxygen carrier in transfusions. On the other hand, an Hr-albumin copolymer restores survival rates to 100% and generally yields biochemical and histological parameters similar to those of glutaraldehyde-polymerized bovine hemoglobin, with the exception of an acid-base imbalance. The latter may be solved by employing an allogeneic albumin as opposed to the human albumin employed in the present study.

## 1. Introduction

Hemoglobin-based oxygen carriers (HBOCs) have been advocated for use in transfusions due to their potential ability to transport oxygen, as opposed to classical plasma expanders currently employed in emergency medicine or surgery. Hemoglobins from various sources, derivatized chemically by various means (often (co) polymerization with glutaraldehyde, but also reticulation with other agents, or derivatization with biocompatible polymers/oligomers to increase the apparent molecular volume and thus prevent extravasation), have been proposed to this end. Bovine hemoglobin polymerized with glutaraldehyde is currently approved for limited human use in two countries, while no other HBOC is currently approved for clinical use anywhere else, despite constant progress in producing less reactive and more stable Hb derivatives [1–12]. Protein-free approaches (oxygen-encapsulating emulsions, fluorinated hydrocarbons, and heme-based dendrimers) have also been reported [9, 13–15].

An alternative we previously proposed for Hb in HBOC has involved hemerythrin (Hr), an oxygen-carrying protein originally extracted from marine worms but also available in recombinant form overexpressed in *E. coli* [15–17]. The active site of Hr contains a nonheme diiron center which compared to hemoglobin heme offers several interesting differences besides the obvious difference in color (Hr is almost colorless compared to Hb, thus not interfering with diagnostic tests that rely on the color of hemoglobin) [16–18]. Thus, the O_2_ molecule when bound to Hb gains a superoxide-like character, which allows it to react rapidly with nitric oxide (since both superoxide and nitric oxide are free radicals) [16–18]; this reaction can lead to serious side effects in terms of arterial tension in HBOC-based transfusions [9, 19–21]. By contrast, O_2_ when bound to Hr gains a peroxide-like character and is essentially inert to nitric oxide [22]. Hr is also, for the same reason, inert to hydrogen peroxide, while the reaction of Hb with peroxides is well-characterized to lead strongly-oxidizinghigh-valent iron and free radicals. Hr is also less reactive to nitrite, compared to Hb [22]. We have reasoned that Hr would have an advantage over Hb if used in HBOC, as being more resistant to oxidative and nitrosative stress agents. In cell culture tests with human cells [23, 24], as well as in top-up animal models (rats injected with low amounts of HBOC, without prior hemorrhage) [25], some Hr‐based HBOCs did, in fact, match or exceed the performance of any of the tested Hb-based HBOC.

We have previously reported an evaluation of a range of physiological parameters in rats injected with small amounts of blood substitute candidates based on hemoglobin, [26] and then also in hemorrhage models. These candidates were generally based on bovine and ovine hemoglobin, polymerized with glutaraldehyde alone or together with serum albumin or with a peroxidase, meant to alleviate oxidative stress [1, 23, 24, 27–29]. Here, two Hr-based HBOC are tested under conditions identical to previous library of Hb-based HBOC in hemorrhagic Wistar rats: glutaraldehyde-polymerized hemerythrin and a hemerythrin copolymer with human serum albumin.

## 2. Materials and Methods

Standard reagents and protein derivatives were of the same sources and stocks as previously described for the top-up and hemorrhagic shock experiments with the very same HBOC as used in the present study (see supporting information for a more detailed description) [26, 30]. The Hr concentrations were 150 *μ*M (calculated per monomer, based on the active UV-vis spectrum of the Hr site as previously described [15, 22, 25]) in the glutaraldehyde-polymerized Hr (pHr) and the glutaraldehyde copolymer of Hr and human serum albumin (pHrHSA) preparations used for treating animals.

Healthy adult male Wistar rats weighing 160 ± 20 g, 24 weeks old, were given free access to standard rat food and water. Rats were kept in a light/temperature controlled room with a light/dark cycle of 12/12 h at 22°C. Animal care and procedures were carried out in accordance with Directive 2010/63/EU and national legislation. The actual project was approved by the Ethical Committee of Babeș-Bolyai University (IRB no. 2012/03.02.2016). At the end the animals were humanely killed by deep anesthesia isoflurane (2%), and they were considered dead when no respiratory and heart activity was recorded. The irreversibility of the phenomena was ensured by axo-atloidian dislocation.

The experiment was performed on male Wistar rats randomly divided into 8 groups, 10 animals each, as previously described [30]. The groups were defined as follows: C (control, not subjected to 30% hemorrhage but with blood extracted for biochemical analyses), H (control hemorrhage, subjected to 30% hemorrhage but not to transfusion), P (subjected to 30% hemorrhage then treated with plasma), pHr (30% hemorrhage, transfused with glutaraldehyde-polymerized hemerythrin in PBS), and pHrHSA (30% hemorrhage, transfused with a copolymer of hemerythrin and human serum albumin). According to previous results and in line with procedures performed by Gutierrez et al. [31] as well as Kowalsky and Brandis (2022) [32] or Hooper and Armstrong (2022) [33], the effusion of 30% of total blood volume has induced a Class II hypovolemic shock that stimulated the rheological behavior of the cardiovascular system with unchanged systolic blood pressure (under physiological conditions).

HBOCs were administered intravenously in a proportional volume with blood effusion during hemorrhage *á jeun* under deep narcosis as previously described [30]. Hemorrhagic status was induced under narcosis, by blood effusion from the retroorbital plexus until the blood volume was 30% of the total blood volume of the rat as previously described [34]. The animals were monitored every 10 minutes for the first two hours after intravenous administration of HBOC, and then every two hours for the next 8 hours, and then again at 24 hours. At the end, the animals were subjected to deep isoflurane narcosis and blood was collected and analyzed as previously described [26, 30]. The values for the C, H, and P groups have also been reported in [30].

The results are presented as mean ± standard deviation of the mean (SD). Biochemical data were subjected to ANOVA followed by Tukey's multiple comparison test when comparing all experimental groups. Histopathological score changes for each tissue were analyzed by using the rank-based nonparametric Kruskal–Wallis test with Dunn's test based on rank for multiple comparisons. In the ANOVA test, *p* < 0.05 was considered statistically significant. Tukey's multiple comparison test was considered statistically significant at *p* < 0.05 and was interpreted as follows: ^*∗*^*p* < 0.05, ^*∗∗*^*p* < 0.01, and ^*∗∗∗*^*p* < 0.001. Significant differences after comparisons across groups were indicated as follow: ^#^*p* < 0.05, ^##^*p* < 0.01, and ^###^*p* < 0.001. For each analysis, *N* (no. of rats or samples) was ten. Statistical analyzes were performed using Graph Pad Prism version 5.0 for Windows, Graph Pad Software, San Diego, CA, USA.

## 3. Results and Discussion

Table 1 shows the survival rates for these experiments, along with the arterial tension (AT) values at the time points. The pHr group shows a reduction in survival rate compared to the untreated hemorrhage group (H), down to 50% from 75%. However, pHrHSA shows full recovery to 100% survival. The AT values immediately after hemorrhage are similar in all three groups. Treatment with pHr or pHrHSA immediately restores 50% of this gap. However, at 24 hours, the pHr group shows a distinct increase in AT compared to the initial values prior to hemorrhage, while in the pHrHSA group, the AT returns to values similar to the prehemorrhage state. From these points of view, pHrHSA appears to be a reasonable candidate for HBOC, while pHr does not.

**Figure 1:**
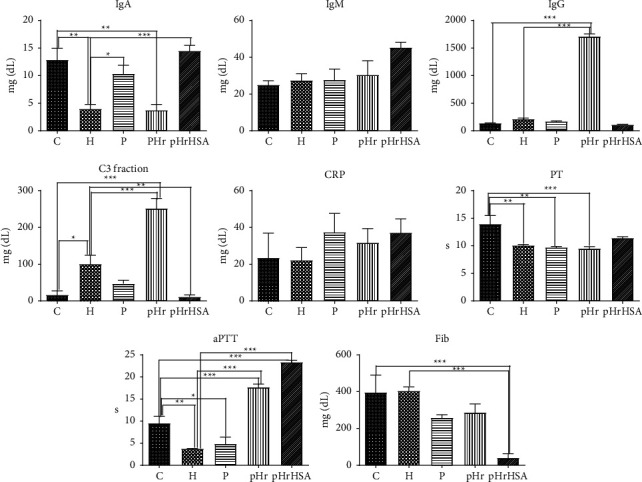
Immunological and clotting parameters. C = control, H = untreated hemorrhage, pHr = hemorrhage treated with polymerized hemerythrin pHr, pHrHSA = hemorrhage treated with hemerythrin-albumin copolymer pHrHSA. Values are expressed as mean ± SD. ^*∗*^significant at *p* < 0.05; ^*∗∗*^significant at *p* < 0.01; ^*∗∗∗*^significant at *p* < 0.001.

Figure 1 shows immunological and clotting parameters after transfusion with pHr or pHrHSA, compared to control groups, not subjected to transfusion (H: loss of 30% blood and C: loss of 5% blood). For IgA, pHr shows levels identical to those of the reference H group, while pHrHSA shows levels identical to those of the control group. For IgM, there are no statistically significant differences between the groups/samples. On the other side, similar immunoglobulin variations in IgA and IgM levels were observed after allicin administration as an immunostimulatory agent, where the IgA and IgM concentration was increased (∼45 mg/dL for IgA and ∼50 mg/dL for IgM) in a dose-dependent manner, and these changes were accepted as beneficial in terms of immunostimulation [35, 36] IgG variation after pHr exposure demonstrated that pHr was recognized as a potent antigen that induced a significant increase in IgG concentration without other immunological imbalances. These reactions also reveal the activation of immunocompetent cells after pHr exposure and a prominent humoral immune response, which is consistent with previous observations according to which IgG amplification is a physiological reaction after blood transfusion [35]. The pHr triggered a strong immune response reflected in elevated plasma levels of IgG accompanied by elevated levels of the C3 complement fraction. This might be a plausible explanation for the high mortality observed in the pHr group, something not seen in previous top-up models, where much lower amounts of pHr were injected [25]. Interestingly, no such problem is seen for pHrHSA, suggesting that HSA in this preparation is efficient at blocking the immunogenic sites on Hr. This again correlates with the fact that the pHrHSA shows a 100% survival rate, improved both over the untreated group (H) as well as over the pHr group. CRP expectedly did not show statistically significant change in any of the groups. For PT, no differences are seen between the hemorrhage group and the treated groups, while for aPTT, both hemerythrin samples show increases over the control and hemorrhage groups. For fibrinogen, pHrHSA shows a distinct drop compared to all other samples. According to some studies [37, 38], high concentrations of human serum albumin form an intravascular sequential adsorption surface for immunoglobulin G, as well as fibrinogen, and their interaction with albumin generates fine fibrils without crosslinking, determining the decrease in measured fibrinogen and IgG in blood.

Tables 2 and S2 show histological data collected from liver, lung, and kidney tissues. Hemorrhage has previously shown to induce slight proliferation of Kupfer cells in the liver and inflammation in the lungs under these conditions, as hypovolemic shock induces congestion through hemodynamic overload. Candidate Hb-based HBOC were also previously shown to generally show worse diagnoses than the hemorrhage group; exceptions were HBOC based on ovine hemoglobin. The performance of pHr and pHrHSA in Table 2 appears similar in terms of damage level to the performance of glutaraldehyde-polymerized Hb, i.e., HBOC was previously approved for limited human use [30].

**Figure 2:**
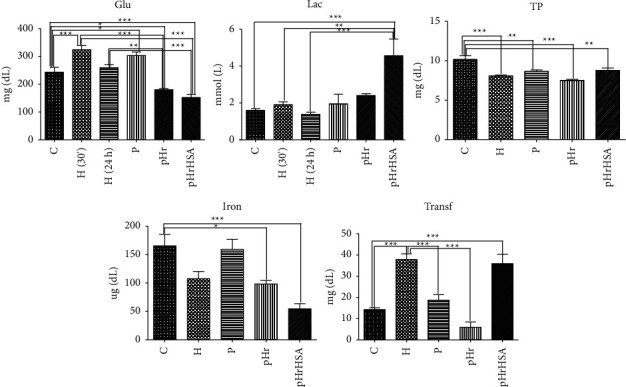
Transferrin, iron, total proteins, glucose, and lactate in control and experimental animals. ^*∗*^Significant at *p* < 0.05; ^*∗∗*^significant at *p* < 0.01; ^*∗∗∗*^significant at *p* < 0.001.

Supporting information Table S1 shows the distribution of iron deposits in the liver. Both pHr and pHrHSA show behavior similar to the control and hemorrhage groups. Furthermore, as shown in supporting information Figure S1, the levels of hematocrit and hemoglobin (measured on samples collected at 24 hours, hence at the same time point as the Table S1) are not statistically different across the samples. The pH of blood, as well as O_2_ and CO_2_ levels are also not affected (Figure S2). pHrHSA shows a drop in BE and in bicarbonate compared to all other samples. This latter observation is matched by urea and creatinine levels, showing a disruption of the renal function, but also by increased lactate levels (cf. Figures 2 and S3, hence lactic acidosis, accompanied by decreased glucose levels). The sodium, potassium, and calcium levels show essentially no variations (only slight decreases in sodium and potassium for pHrHSA, cf.  Figure S4). The lactic acidosis seen with pHrHSA may be ascribed to the presence of HAS, as pHr shows no statistically significant differences compared to the control group.

Figure 2 shows total protein, transferrin, iron, glucose, and lactate levels. Iron and transferrin levels are slightly affected in Hr samples, but remain at values similar to the control or the untreated hemorrhage group. The total protein level does not change in a statistically significant manner after transfusion with pHr or pHrHSA. The glucose levels are distinctly lower in the Hr groups and slightly more in pHrHSA compared to pHr; this is mirrored by increase in lactate. The changes in the pHrHSA group may be linked to the fact, previously commented upon, that serum albumin from other organisms may cause imbalances itself, beyond the effect of the oxygen-carrying protein in the HBOC. If so, an Hr copolymer with rat albumin would perform better than Hr-HAS, which was previously observed for Hb-albumin copolymers [30].

## 4. Conclusions

Two hemerythrin-basedglutaraldehyde-polymerized HBOCs were analyzed in shock models (30% blood loss, treated by transfusion with polymerized Hr or with a Hr-HAS copolymer). A notable immune response was observed for pHr in terms of IgG (but not IgM or IgA), while no such response was observed for the copolymer pHrHSA. Consistent with this, pHr transfusion led to a decrease in survival rate compared to the untreated hemorrhage group, while pHrHSA restored the survival rate to 100%. Both pHr and pHrHSA induced limited tissular damage to levels similar to those observed for glutaraldehyde-polymerized bovine hemoglobin. Acidosis was detected with both of the Hr-based preparations (especially for pHrHSA), while other physiological parameters remained within normal limits. To conclude, Hr alone cannot form the basis of an efficient HBOC; however, when conjugated with other large molecules (e.g., serum albumin) an acceptable HBOC might be generated if the acid-base problem seen for pHrHSA can be solved.

## Figures and Tables

**Table 1:** Average blood pressure and survival rates at 24 hours.

Group	Initial	Shock	Treatment	Final	Survival (%)^b^
C	107 ± 25	NA^a^	NA^a^	115 ± 11	100
H	101 ± 25	65 ± 15^*∗∗*^	NA^a^	89 ± 26	75
pHr	121 ± 27	85 ± 19^*∗∗*^	100 ± 40	152 ± 21^*∗*,###^	50
pHrHSA	104 ± 9	67 ± 13^*∗∗∗*^	84 ± 36^*∗*^	108 ± 6^###^	100

^a^Not applicable; ^b^survivors after administration of treatment (at 24 hours); values are expressed as mean ± SD; (^*∗*^) significant at *p* < 0.05, (^*∗∗*^) significant at *p* < 0.01, (^*∗∗∗*^) significant at *p* < 0.001 as compared with “Initial” values; (^#^) significant at *p* < 0.05, (^##^) significant at *p* < 0.01; (^###^) significant at *p* < 0.001 as compared with “Shock” values. The *t*-test was applied.

**Table 2:** Summary of histological findings of transfusion experiments in the present study.

Groups	Liver					Lungs		Kidneys							
	GVD	DG	N	S	K	ED	S	N	GVD	DG	TL	S	M	USD	TD
C	—	—	—	—		—	—	—	—	—	—	—	—	—	—
H	—	—	—	—	+	—	—	—	—	—	—	—	—	—	+
P	—	VD++	—	—		—	—	—	—	—	—	—	+/−	—	—
pHr	+++		++	—		—	—	—	—	VD+	—	—	—	—	—
pHrHSA	++	VD++	—	—		—	—	—	+/−		++	—	—	—	++

GVD: granular and vacuolar degeneration, DG: degeneration, VD/GD: vacuolar degeneration/granular degeneration, N: necrosis, S: stasis, K: Kupffer cells, ED: edema, TL: tubular lesions, M: mesangial proliferation, USD: urinary space dilation, TD: tubular dilation; blank: no changes/not present, +/−: slight/absent changes, +: slight changes, ++: moderate changes, and +++: prominent changes.

## Data Availability

Supporting information data are available on hematocrit, hemoglobin, acid-base parameters, renal function parameters, blood ion concentrations, iron deposit evaluation. Furthermore, primary data for all tables and figures in the manuscript are available upon request from the authors.

## Supplementary Materials

Supplementary Materials A file with supporting information is available, including data on hematocrit, hemoglobin (Figure S1), acid-base parameters (Figure S2), renal function parameters (Figure S3), blood ion concentrations (Figure S4), characteristics of the tested materials (text and Table S1), and evaluation of iron deposit evaluation (Table S2).
