# Supplementary material for: Glutaraldehyde-Polymerized Hemerythrin: Evaluation of Performance as an Oxygen Carrier in Hemorrhage Models
Source: Bioinorg Chem Appl. 2022 Dec 30;2022:2209101. doi: 10.1155/2022/2209101 (PMC9822766; doi:10.1155/2022/2209101)
Supplement: Supplementary Materials — A file with supporting information is available, including data on hematocrit, hemoglobin (Figure S1), acid-base parameters (Figure S2), renal function parameters (Figure S3), blood ion concentrations (Figure S4), characteristics of the tested materials (text and Table S1), and evaluation of iron deposit evaluation (Table S2). [file 2209101.f1.docx]

Supporting Information

**Glutaraldehyde-polymerized hemerythrin: evaluation of performance as an oxygen carrier in hemorrhage models**

Anca D. Stoica^1,2+^, Vlad-Al. Toma^1,3+^, Ioana Roman^3^, Bogdan Sevastre^4^, Florina Scurtu^5^, Radu Silaghi-Dumitrescu^5^*

*^1^ Department of Molecular Biology and Biotechnology, Babeș-Bolyai University, Cluj-Napoca, Romania;*

*^2^National Institute for Research and Development of Isotopic and Molecular Technologies, 400293 Cluj-Napoca, Romania*

*^3^Institute of Biological Research, Cluj-Napoca 400113, branch of NIRDBS Bucharest, Romania*

*^4^Department of Pathophysiology, University of Agricultural Sciences and Veterinary Medicine, Cluj-Napoca, 400372, Romania*

*^5^Faculty of Chemistry and Chemical Engineering, Babeș-Bolyai University, Cluj-Napoca 400028, Romania*

*Correspondence to Radu Silaghi-Dumitrescu:* [radu.silaghi@ubbcluj.ro](mailto:radu.silaghi@ubbcluj.ro)

^+^ authors with equal contributions

**Detailed procedure for pHr and pHrHSA purification**

Overexpression, purification and polymerization of recombinant *P. gouldii* Hr was performed as described in previous studies.[1] BL21 (DE3) *Escherichia coli* cells, suitable for transformation and protein expression were subjected to heat shock with pDK4–1 plasmid containing the gene of *P. gouldii* concentration basis, determined spectrophotometrically with Hr. Transformed cells, resistant to ampicillin, were selected on LB/amp agar plates. A single colony was grown overnight in 50 mL LB/amp medium. This pre-culture was then transferred in 1 L of LB/amp medium and incubated at 37 °C with shaking at 250 rpm. When suitable cell density was reached, (OD_600_: 0.8 - 1, approximately 2.5 h), expression was induced with 0.4 mM isopropyl-b-D-thiogalactoside. Cells were further cultured for 5 - 8 h at 37 °C and then overnight at 25°C, then separated by centrifugation, resuspended in a minimal volume of Tris/NaCl pH 7.4 and lysed by sonication. A vigorous centrifugation succeeded and the pellet, containing apoHr in the form of inclusion bodies, was dissolved overnight in cca. 12 mL GndCl/b-ME. A subsequent centrifugation and the dilution of guanidinic supernatant with a 10-fold volume of Tris/NaCl which was added dropwise resulted in renatured, insoluble apoHr of white color. The suspension was centrifuged, the pellet was redissolved in 12 mL of Gnd/b-ME, and the apoHr was renatured again, by very slow addition (over 6–10 h) of 10 volumes of deoxygenated Tris/NaCl pH 7.4, in the presence of 0.1 g of ferrous ammonium sulfate, under anaerobic conditions and continuous stirring, at 4 °C. In this way, soluble deoxyHr was obtained, along with unincorporated FeII and insoluble apoHr. The apoHr was removed by centrifugation, the unicorporated FeII was oxidized by 1 h stirring in air, and the resulted insoluble FeIII was removed by dialysis. Finally, excess GndCl/b-ME was removed by dialysis against Tris/NaCl pH 7.4. After these steps, the presence of Hr (mainly in its oxy form) could be verified by its characteristic UV–vis absorption. The Hr was converted to its more stable met form by the addition of a few crystals of potassium ferricyanide and overnight stirring at 4 °C. Ferrocyanide and excess ferricyanide were removed by dialysis against PBS, and the protein was concentrated in an Amicon ultrafiltration unit (Millipore Corp., Billerica, MA). If the UV–vis absorption spectrum of the resulting metHr indicated the presence of any residual ferricyanide (A330 nm/A380nm absorbance ratio >1.2), then the protein solution was passed over a PD-10 desalting column (GE Healthcare, Chalfont, UK). The A280 nm/A380 nm absorbance ratio of the final product was 5.8, which is lower by one unit compared with the material used in our previous report on derivatized hemerythrin (Mot et al. 2010), suggesting a slightly higher purity. The average yield of recombinant metHr was 40 mg protein/L of culture medium. Purified metHr was stored in PBS at -20 °C. (Co)polymerization reactions were carried out in PBS at a 0.15 mM constant concentration of metHr (protein monomer concentration basis, determined spectrophotometrically with ε(350 nm) = 6400 M^-1^cm^-1^. A 0.75 mM HSA stock solution was prepared by dissolving 0.05 g HSA (Sigma-Aldrich, Chicago, IL) in 950 µl PBS. If necessary, successive dilutions of this stock solution were used. GL-cross-linking presumed the reaction of the two (–CHO) carbonyl groups of GL with the (–NH_2_) amino groups of the proteins. The concentration of lysines was estimated as the molar concentration of protein multiplied by the number of lysine residues/(monomeric) unit (11 in case of Hr, 59 in case of HSA. GL stock solutions at 100mM were prepared from a 25% (2.65 M) solution (Sigma-Aldrich, Chicago, IL). The concentration of GL used for cross-linking was between 4 mM. The steps of derivatization were very similar to those described in a previous study.[2] GL-cross-linking lasted for 2 h at 4 °C with continuous stirring. The reaction was stopped by addition of a 2-fold molar excess (with respect to GL concentration) of sodium borohydride from a freshly prepared, 1 M stock solution. Twenty minutes after the addition of the borohydride, 1/15 volume of 1 M Tris-HCl, pH 7.4 was added, and the solution was allowed to react for a further 20 min. Removal of excess reagents and/or buffer exchange was accomplished using dialysis. The protein was then purified chromatographically as described below.

Electrophoresis was carried out in 15% polyacrylamide gels at 200 V limiting voltage and 25 mA limiting current. Spectroscopic measurements were performed on a Cary 50 (Varian, Inc., Palo Alto, CA) UV–vis spectrophotometer. For gel-filtration size exclusion chromatography analyses, an FPLC System was employed, with a Superdex^TM^ 200 5/150 GL column (GE Healthcare, Chalfont, UK) with Tris/NaCl as the elution buffer. Anion exchange chromatography was performed on an 1mL HiTrap Q FF column (GE Healthcare, Chalfont, UK) using a 20 mM Tris-HCl, pH 7.5 buffer as the mobile phase and a gradient of 20 column volumes was applied to elute the components (20 mM Tris, 1 M NaCl, pH 7.5 elution buffer). The final product was centrifuged at 12000 – 14000 rpm for 40 minutes to eliminate any particulate matter (occasionally recombinant proteins such as Hr may precipitate after freeze-thaw cycles), filtered with a micrometer-level filter (0.20 µM, Chromafil , Macherey-Nagel) and then purified over a fresh LPS column (High-Capacity Endotoxin Removal Column,Thermo Scientific; the resin was activated overnight using 0.2 N NaOH or in 1-2 hours using 0.2N NaOH in 95% ethanol, then washed with 2 M NaCl and then with endotoxin-free ultrapure water); the endotoxin concentration of the HBOCs was < 0.1 ng/mL which is accepted as a safe limit for recombinant proteins all of which are standard procedures in such experiments. Table S1 shows the characteristic parameters of the Hr polymer and Hr-HAS copolymer.

**Table S1. Hemerythrin-derived samples inoculated in rats in the present study (from** [3]**).**

| **Parameters** | **pHr** | **pHrHSA** |
| --- | --- | --- |
| Description | Glutaraldehyde-derivatized Hr | Hr-HSA copolymer with glutaraldehyde |
| MW [kDa] | 64-750 | 64-750 |
| Concentration [mM monomer Hr] | 0.5 | 0.5 |
| P_50_ [mmHg] | 14-17 | 24 |
| pH | 7.4 | 7.4 |
| Buffer | PBS | PBS |
| t_1/2_ (autooxidation) [min] | 360-790 | 390 |
| Conformational state | polymer | copolymer |
| Viscosity (centipoise) at 37 C | <3 | <3 |
| Colloid osmotic pressure (mmHg) | <60 | <60 |

**Figure S1.** Hematocrit and hemoglobin values. C = control, H(30’) = hemorrhage untreated after 30 minutes, H(24h) = hemorrhage untreated after 24 hours, P = hemorrhage treated with plasma, pHr = hemorrhage treated with polymerized hemerythrin pHr, pHrHSA = hemorrhage treated with the hemerythrin-albumin copolymer pHrHSA.

* Significant at *p* < 0.05; ** Significant at *p* < 0.01; *** Significant at *p* < 0.001

**Figure S2.** Acid base equilibrium of control and experimental animals. C = control, H(30’) = hemorrhage untreated after 30 minutes, H(24h) = hemorrhage untreated after 24 hours, P = hemorrhage treated with plasma, pHr = hemorrhage treated with polymerized hemerythrin pHr, pHrHSA = hemorrhage treated with the hemerythrin-albumin copolymer pHrHSA.

* Significant at *p* < 0.05; ** Significant at *p* < 0.01; *** Significant at *p* < 0.001 (compared with Control)

**Figure S3.** Renal function parameters of control and experimental animals. C = control, H(30’) = hemorrhage untreated after 30 minutes, H(24h) = hemorrhage untreated after 24 hours, P = hemorrhage treated with plasma, pHr = hemorrhage treated with polymerized hemerythrin pHr, pHrHSA = hemorrhage treated with the hemerythrin-albumin copolymer pHrHSA.

* Significant at *p* < 0.05; ** Significant at *p* < 0.01; *** Significant at *p* < 0.001

**Figure S4.** Blood ion concentration of control and experimental animals. C = control, H(30’) = hemorrhage untreated after 30 minutes, H(24h) = hemorrhage untreated after 24 hours, P = hemorrhage treated with plasma, pHr = hemorrhage treated with polymerized hemerythrin pHr, pHrHSA = hemorrhage treated with the hemerythrin-albumin copolymer pHrHSA.

* Significant at *p* < 0.05; ** Significant at *p* < 0.01; *** Significant at *p* < 0.001

**Table S2. Iron deposits evaluation by Pearls staining in liver.** The evaluation of the histochemical reaction was done by Grover et al. (2015) method as follows: **+/-**, slight to absent reaction, **+,** slight reaction, **++**, moderate reaction, **+++**, intense reaction. C = control, H = hemorrhage untreated after 24 hours, pHr = hemorrhage treated with polymerized hemerythrin pHr, pHrHSA = hemorrhage treated with the hemerythrin-albumin copolymer pHrHSA.

| **Group** | **Reaction** | **Group** | **Reaction** |
| --- | --- | --- | --- |
| **C** | **++** | pHr | -/+ |
| **H** | **+** | pHrHSA | + |

**References**:

1. Arkosi, M.; Scurtu, F.; Vulpoi, A.; Silaghi-Dumitrescu, R.; Kurtz, D.; Kurtz Jr., D.M. Copolymerization of Recombinant P. gouldii Hemerythrin with Human Serum Albumin for Use in Blood Substitutes. *Artificial Cells Blood Substitutes and Biotechnology* **2017**, *45*, 218–223.

2. Mot, A.C.; Roman, A.; Lupan, I.; Kurtz Jr., D.M.; Silaghi-Dumitrescu, R. Towards the development of hemerythrin-based blood substitute. *Protein Journal* **2010**, *29*, 387–393.

3. Toma, V.A.; Farcas, A.D.A.D.; Roman, I.; Sevastre, B.; Hathazi, D.; Scurtu, F.; Damian, G.; Silaghi-Dumitrescu, R. In vivo evaluation of hemerythrin-based oxygen carriers: Similarities with hemoglobin-based counterparts. *Int J Biol Macromol* **2017**, *107*, 1422–1427.
